# PT-112 Induces Mitochondrial Stress and Immunogenic Cell Death, Targeting Tumor Cells with Mitochondrial Deficiencies

**DOI:** 10.3390/cancers14163851

**Published:** 2022-08-09

**Authors:** Ruth Soler-Agesta, Joaquín Marco-Brualla, Martha Minjárez-Sáenz, Christina Y. Yim, Marta Martínez-Júlvez, Matthew R. Price, Raquel Moreno-Loshuertos, Tyler D. Ames, José Jimeno, Alberto Anel

**Affiliations:** 1 Department of Biochemistry and Molecular and Cell Biology, Aragón Health Research Institute (IIS-Aragón), University of Zaragoza, 50009 Zaragoza, Spain; 2 Institute for Biocomputation and Physics of Complex Systems, University of Zaragoza, 50018 Zaragoza, Spain; 3 Promontory Therapeutics Inc., New York, NY 10019, USA

**Keywords:** PT-112, cancer cell death, mitochondrial ROS, CoQ10, immunogenic cell death, ICD, HIF-1α

## Abstract

**Simple Summary:**

PT-112 is a novel pyrophosphate–platinum conjugate under Phase 1/2 clinical development for the treatment of several tumor types. In this study, using mouse tumor cells with well-characterized mitochondrial and metabolic status, we investigated the mechanisms underlying PT-112’s cancer cell death effects. Our results showed that cells with defective mitochondria were more sensitive to PT-112 when compared to cells with normal mitochondrial function. Moreover, PT-112 induced tumor cell death in those sensitive cells through non-conventional mechanisms, including increased mitochondrial stress, free radical generation and immunogenic cell death, a form of cell death that elicits an immune response. Taken together, the present findings suggest the potential for predictors of PT-112 sensitivity in the clinical setting on the basis of metabolic function.

**Abstract:**

PT-112 is a novel pyrophosphate–platinum conjugate, with clinical activity reported in advanced pretreated solid tumors. While PT-112 has been shown to induce robust immunogenic cell death (ICD) in vivo but only minimally bind DNA, the molecular mechanism underlying PT-112 target disruption in cancer cells is still under elucidation. The murine L929 in vitro system was used to test whether differential metabolic status alters PT-112’s effects, including cell cytotoxicity. The results showed that tumor cells presenting mutations in mitochondrial DNA (mtDNA) (L929dt and L929^dt^ cybrid cells) and reliant on glycolysis for survival were more sensitive to cell death induced by PT-112 compared to the parental and cybrid cells with an intact oxidative phosphorylation (OXPHOS) pathway (L929 and dt^L929^ cybrid cells). The type of cell death induced by PT-112 did not follow the classical apoptotic pathway: the general caspase inhibitor Z-VAD-fmk did not inhibit PT-112-induced cell death, alone or in combination with the necroptosis inhibitor necrostatin-1. Interestingly, PT-112 initiated autophagy in all cell lines, though this process was not complete. Autophagy is known to be associated with an integrated stress response in cancer cells and with subsequent ICD. PT-112 also induced a massive accumulation of mitochondrial reactive oxygen species, as well as changes in mitochondrial polarization—only in the sensitive cells harboring mitochondrial dysfunction—along with calreticulin cell-surface exposure consistent with ICD. PT-112 substantially reduced the amount of mitochondrial CoQ10 in L929 cells, while the basal CoQ10 levels were below our detection limits in L929dt cells, suggesting a potential relationship between a low basal level of CoQ10 and PT-112 sensitivity. Finally, the expression of HIF-1α was much higher in cells sensitive to PT-112 compared to cells with an intact OXPHOS pathway, suggesting potential clinical applications.

## 1. Introduction

PT-112 (R,R-1,2 cyclohexanediamine-pyrosphosphato-platinium (II)) is a novel compound consisting of a stable pyrophosphate conjugated to a diaminocyclohexane-platinum (Pt) ring [1]. In comparison to traditional platinum-containing agents, it shows substantially reduced DNA binding with minimal acute renal toxicities and neurotoxicity, as shown in early in vitro studies in yeast models, human ovarian cancer cell lines, and in vivo models [2,3,4]. PT-112 has demonstrated anti-tumor activity in mice bearing various murine and human tumors at sub-toxic doses [2,3,5,6,7], robust induction of immunogenic cell death (ICD) [7], and systemic biodistribution to multiple tissues and organs, including kidney, lung and liver, with the highest concentrations reached in bone [5]. These observations may be explained at least in part by PT-112’s organic pyrophosphate moiety, which is chemically similar to a bisphosphonate. Bisphosphonates can bind to mineral bone surfaces [8] and are generally used in oncology for the management of skeletal complications and/or bone metastases. In addition, some bisphosphonates have been shown to exhibit anti-cancer properties by targeting specific enzymes of the mevalonate pathway [9,10]. Specifically, the nitrogen-containing bisphosphonates inhibit farnesyl pyrophosphate synthase (FPPS), a key enzyme of the mevalonate pathway [11,12].

Preclinical findings with PT-112 provided a rationale for clinical development, and Phase I data reported from the first-in-human study of PT-112 monotherapy in a heavily pretreated population with solid tumors showed favorable tolerability and safety profiles, as well as evidence of efficacy, including prolonged responses and tumor control in different tumor types, such as non-small cell lung cancer, small cell lung cancer and thymoma (ClinicalTrials.gov ID: NCT02266745), [13]. Notably, clinical and serologic responses to the monotherapy in a small sub-population of late-stage, metastatic castration-resistant prostate cancer (mCRPC) patients were observed, and a median overall survival of 15.1 months was reached [13,14]. In addition, in a combination study with PD-L1 immune checkpoint inhibitor avelumab (ClinicalTrials.gov ID: NCT03409458), PT-112 was shown to be feasible with a lack of overlapping toxicities, and preliminary evidence of efficacy included long-lasting objective remissions and tumor control in mCRPC and other solid tumors [13,14,15]. PT-112 was also shown to have biological activity in mouse models of multiple myeloma [5] and was the subject of a Phase I clinical trial in heavily pretreated multiple myeloma patients (ClinicalTrials.gov ID: NCT03288480), where monotherapy activity has been reported [16]. Phase II studies of PT-112 are currently ongoing.

Along with the progress made in the clinical setting, a major effort has been made in translational studies to discern the mechanisms underlying PT-112 target disruption. In previous work from our laboratory, we characterized the L929dt cell subline derived from the transformed fibroblast cell line L929 [17]. L929dt cells lost matrix attachment and MHC-I expression and showed a lower capacity to generate energy through OXPHOS, as well as decreased respiratory capacity compared to parental L929 cells. These defects correlated with mtDNA mutations in L929dt cells at the *ND2* subunit of complex I and were accompanied by a substantial glycolytic shift and higher in vivo tumorigenic and metastatic potential than the parental cell line. We also demonstrated that mitochondrial mutations are responsible for the aggressive tumor phenotype by generating cybrid cells with L929dt mitochondria in L929 nuclear background (L929^dt^ cells), which reproduced all L929dt properties. Remarkably, cybrid cells with parental L929 mitochondria in the L929dt nuclear background (dt^L929^ cells) recovered the complete parental L929 phenotype [18].

This cellular system, with distinct glycolytic and metabolic phenotypic properties due to defined mitochondrial DNA mutations, was used to explore whether differential metabolic and mitochondrial status alters PT-112-driven effects and cytotoxicity in cells. In addition, we investigated the mechanism underlying cell death induced by PT-112 in this cellular system, connecting to known PT-112 immunogenic cell death potential, and tried to relate it to the expression of specific biomarkers such as HIF-1α.

In the present work, we demonstrate that PT-112 does not kill cells in this L929 panel exclusively by apoptosis or necroptosis but rather by initiating an unresolved autophagy process and causing mitochondrial stress and membrane disruption. Cells that have an intact OXPHOS pathway can cope with this PT-112-induced stress, but in cells that have an extreme glycolytic and hypoxic phenotype as indicated by HIF-1α expression, exposure to PT-112 is associated with massive mitochondrial ROS generation and cell death. These observations suggest that PT-112’s mechanism of action may be selective to cancer metabolic processes, with the potential for clinical applications of PT-112 in metabolically aggressive cancers.

## 2. Materials and Methods

### 2.1. Cell Culture

Mouse fibroblast cell lines L929 and L929-derived, “detached” cells (L929dt) were routinely cultured in high glucose DMEM medium with GlutaMAX (Life Technologies, Paisley, UK) supplemented with 10% of fetal calf serum (FCS; Sigma, St. Louis, MO, USA), penicillin (1000 U/mL) and streptomycin (10 mg/mL) (PanBiotech, Aidenbach, Germany) at 37 °C and 5% CO_2_ using standard procedures. The cybrid cell lines L929^dt^ and dt^L929^ were obtained as previously described [18] and cultured with the identical medium as parental cells. For L929-ρ^0^ cells, complete DMEM medium was also supplemented with pyruvate (100 µg/mL) and uridine (50 µg/mL).

### 2.2. Cell Viability Assays

Relative cell growth was measured using a modified Mossman method for microplates. Briefly, 3 × 10^4^ cells were seeded per well in a 96-well flat-bottom plate and incubated with increasing concentrations of PT-112 or cisplatin (2, 6 and 10 µM) for 24–72 h at 37 °C. Then, 10 µL of a 5 mg/mL MTT solution was added to each well and incubated for 3 h. During the incubation time, viable cells reduced the MTT solution to insoluble purple formazan crystals, which were subsequently solubilized with isopropanol and 0.05 M HCl mixture, and the absorbance was measured in a microplate reader (Dynatec, Pina de Ebro, Spain). The MTT method in mitochondria-defective cell lines has been used in other studies, as MTT reduction is performed not only by mitochondrial enzymes but also by other cellular oxidoreductases [19,20]. Control cell lines were included in each growth experiment.

### 2.3. Cytotoxicity Assays and Cell Death Quantification

Cytotoxicity assays were performed as follows: 100 µL aliquots of 3 × 10^4^ cells were seeded per well in a 96-well plate, and 10 µM of PT-112 or cisplatin were added and incubated for 24–72 h at 37 °C. Cell death was analyzed using a FACSCalibur flow cytometer (BD, Biosciences) after incubation with annexin-V-FITC and 7-AAD (BD Biosciences, Madrid, Spain) in annexin binding buffer (140 mM NaCl, 2.5 mM CaCl_2_, 10 mM HEPES/NaOH, pH 7.4) for 10 min.

### 2.4. ROS Production and Mitochondrial Membrane Potential Measurement

Total ROS production and mitochondrial membrane potential were simultaneously measured using a FACScalibur flow cytometer (BD Biosciences). Pretreated cells with PT-112 were incubated with DiOC_6_ at 20 nM (Molecular Probes, Madrid, Spain) and DHE at 2 µM (Molecular Probes, Madrid, Spain) for 30 min at 37 °C. For mitochondria-specific ROS production, cells were incubated with MitoSOX™ (5 µM, ThermoFisher, Rockford, IL, USA) for 30 min at 37 °C.

### 2.5. Apoptosis and Necroptosis Inhibition Assays

In total, 3 × 10^4^ cells were seeded in a 96-well plate and incubated with a pan-caspase inhibitor Z-VAD-fmk (50 µM, MedChem Express, Monmouth Junction, NJ, USA) and/or RIPK-1 inhibitor necrostatin-1 (30 µM, MedChem Express, Monmouth Junction, NJ, USA) for 1 h. Next, cells were treated with 10 µM of PT-112 and incubated for 48 h at 37 °C. Both inhibitors were refreshed in their corresponding well after 24 h. Finally, cell death was assessed using flow cytometry after incubation with annexin-V-FITC and 7-AAD for 10 min.

### 2.6. Analysis of Caspase-3 Activation

Caspase-3 activation was measured using a FITC-labelled antibody against cleaved caspase-3 (BD Pharmingen™, Madrid, Spain). Cells pretreated with 10 µM of PT-112 were fixed with 4% paraformaldehyde solution for 15 min at 4 °C. Then, cells were washed with PBS buffer, permeabilized using a 0.1% saponin dilution supplemented with 5% fetal bovine serum and incubated for 15 min at room temperature (RT). After washing them, samples were incubated with the antibody for 30 min at RT and analyzed by flow cytometry.

### 2.7. Cyto-ID^®^ Analysis and Autophagosome Formation Measurement

For autophagy analysis, the autophagosome formation after treatment with PT-112 was evaluated using a Cyto-ID^®^ probe (Enzo Life Sciences, Farmingdale, NY, USA). Cells pretreated with 10 µM of PT-112 were incubated with 1 µL of Cyto-ID^®^ dye reagent for 30 min at 37 °C. Subsequently, cells were washed with PBS buffer and analyzed by flow cytometry. For autophagy-positive controls, cells were treated with 1 µM of rapamycin at least 12 h before the analysis.

### 2.8. Calreticulin (CRT) Surface Expression Measurement

CRT surface expression upon incubation with PT-112 (24–72 h) was analyzed by flow cytometry. Cells treated with PT-112 were incubated with primary rabbit antibody (Abcam, #AB2907) at 1:700 dilution and at 4 °C for 1 h. Then, cells were washed with PBS and incubated simultaneously with secondary goat anti-rabbit IgG antibody conjugated with Alexa Fluor488^®^ (Invitrogen, #A11034) and 7-AAD. To rule out non-specific interactions, untreated cells were incubated only with secondary antibody. 7-AAD-positive cells were excluded from the analysis.

### 2.9. Immunoblot Analysis

A total of 5 × 10^6^ cells were lysed with 100 µL of 1× lysis buffer (1% Triton-X-100; 150 mM NaCl; 50 mM Tris/HCl pH 7,6; 10% *v/v* glycerol; 1 mM EDTA; 1 mM sodium orthovanadate; 10 mM sodium pyrophosphate; 10 μg/mL leupeptin; 10 mM sodium fluoride; 1 mM methyl phenyl sulfide, Sigma, St. Louis, MO, USA) for 30 min on ice. The mixture was ultra-centrifugated at 12,000 rpm for 20 min at 4 °C. The protein concentration in the supernatant was analyzed using the BCA assay (Thermo Fisher, Rockford, IL, USA) and was mixed with 3× lysis buffer (SDS 3% *v*/*v*; 150 mM Tris/HCl; 0.3 mM sodium molybdate; 30% *v/v* glycerol; 30 mM sodium pyrophosphate; 30 mM sodium fluoride; 0.06% *p*/*v* bromophenol blue; 30% *v/v* 2-mercaptoethanol, all purchased from Sigma, St. Louis, MO, USA). Protein separation was performed using SDS-PAGE (6% or 12% polyacrylamide gel), and proteins were transferred to nitrocellulose membranes using a semi-dry electro-transfer (GE Healthcare, Chicago, IL, USA). Membranes were blocked with TBS-T buffer (10 mM Tris/HCl, pH 8.0; 120 mM NaCl; 0.1% Tween-20, 0.1 g/L thimerosal, Sigma, St. Louis, MO, USA) containing 5% skimmed milk. Protein detection was performed by western blot using specific antibodies against p62 (Santa Cruz, SC-28359), LC3BI/II (Sigma, L7543) and HIF-1α (Novus, NB100–479) that were incubated overnight at 4 °C in agitation. Anti-rabbit or anti-mouse secondary antibodies labeled with peroxidase (Sigma, A9044) were incubated for 1 h at RT. Blots were analyzed with Pierce ELC Western Blotting Substrate (Thermo Scientific, Rockford, IL, USA) using an Amersham Imager 680 (GE Healthcare Life Sciences). Protein expression was quantified by densitometry using ImageJ software. β-actin or tubulin levels were used as a reference to normalize data (Cell Signaling, 3700).

### 2.10. Coenzyme Q Quantification

Mitochondria were isolated from cultured cell lines as previously described [21] with small modifications [22]. A mitochondrial pellet was immediately frozen in liquid nitrogen under anaerobic conditions. Then, lipids extraction and CoQ determination were performed as described previously [23]. Briefly, mitochondrial samples (0.6 mg of mitochondrial protein for each measurement) were lysed with 1% SDS and vortexed for 1 min. Then, a mixture of ethanol:2-propanol (95:5) was added and the samples vortexed again for 1 min. To recover CoQ, 5 mL of hexane was added, and the samples were centrifuged at 1000× *g* for 5 min at 4 °C. The upper phases from two extractions were recovered and dried using a rotary evaporator. Lipid extracts were suspended in 1 mL ethanol, dried in a speed-vac and stored at −20 °C. Samples were resuspended in a suitable volume of ethanol prior to HPLC injection. Lipid components were separated with an Alliance HPLC system (Waters) equipped with a 2707 autosampler and 2996 photodiode array detector, with HSST3 column (4.6 × 150 mm, 3.5 µm, Waters), preceded by a pre-column (4.6 × 20 mm, 3.5 µm, Waters), with a flow rate of 1 mL/min and a mobile phase containing 40:60 methanol/2-propanol. Commercial CoQ10 (Sigma) was used as an internal standard to detect the CoQ10 peak in the samples.

### 2.11. Statistical Analysis and Data Processing

Statistical analysis was performed using GraphPad Prism8 (GraphPad Software Inc., San Diego, CA, USA). For quantitative variables, results are shown as mean ± standard deviation (SD). Statistical significance was evaluated using Student’s *t*-test, and differences were considered significant when *p* < 0.05. Data obtained by flow cytometry were analyzed using FlowJo 10.0.7 (Tree star Inc., San Francisco, CA, USA).

## 3. Results

### 3.1. Cell Growth Inhibition by PT-112 and Cisplatin in L929, L929dt and Cybrid Cells

**Figure 1:**
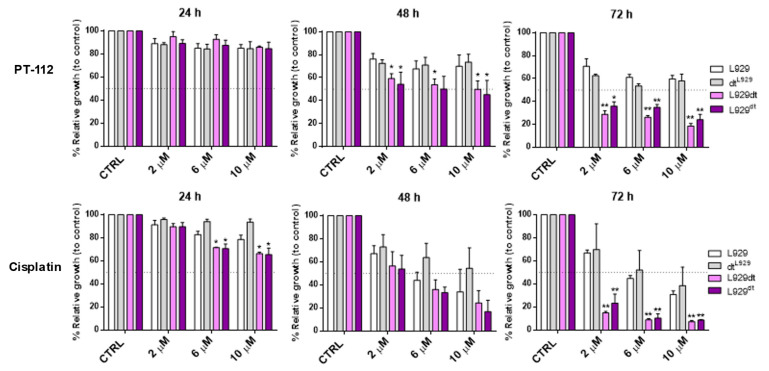
Cell growth analysis after treatment with PT-112 or cisplatin, as indicated. Cells were treated with increasing concentrations of PT-112 or cisplatin, incubated for 24–72 h, and cell growth was measured by the MTT assay. Results were expressed as the percentage of relative growth compared to control, untreated cells (CTRL) ± SD of at least 2 independent experiments performed in duplicate. Statistical significance represents those values in which cell growth inhibition by the drugs was higher in a given cell type as compared with parental L929 cells. *, *p* < 0.05; **, *p* < 0.01.

First, we compared the sensitivity of L929, L929dt and cybrid cells to PT-112 and to cisplatin, a Pt-based chemotherapeutic agent whose mechanism of action is known to involve DNA damage and apoptosis induction [24]. All cell lines were treated with increasing concentrations of PT-112 or cisplatin (2, 6 and 10 µM) and incubated for 24–72 h at 37 °C. The concentrations used are compatible with active levels achieved during well-tolerated in vivo experiments [13,14]. Cell growth was assessed by the MTT reduction method. As shown in the upper panel of Figure 1, PT-112 inhibited cell growth in a time-dependent manner, as a clear decrease in cell growth was not observed until 48 h of exposure. We also observed that the cell lines with mtDNA mutations, L929dt and the L929^dt^ cybrid, were noticeably more sensitive to PT-112 than L929 cells and the dt^L929^ cybrid. This difference was statistically significant at 48 h and was more pronounced at 72 h, where the growth of glycolytic cells was inhibited by around 80% at the highest concentration. On the contrary, limited growth inhibition was observed in the OXPHOS-competent L929 and dt^L929^ cybrid cells at 48 h (i.e., <40% inhibition at the higher concentration used), and the degree of inhibition did not further increase with increased exposure time.

For cisplatin treatments (Figure 1, bottom panel), we observed a significant effect on cell growth starting at 24 h. At 48 h, cisplatin inhibited the growth of all cell lines, with no statistically significant differences among them, contrary to those observed for PT-112. At 72 h, cisplatin-induced effects on growth were more pronounced in the glycolytic cells, although dose-dependent effects were also seen in the non-glycolytic cell lines (i.e., 95% inhibition in L929dt and L929^dt^ cells and 70% inhibition in L929 and dt^L929^ cells at 10 µM cisplatin).

These data demonstrate that PT-112 has a marked selectivity for tumor cells with defective mitochondria (i.e., those exhibiting a glycolytic phenotype), suggesting a relationship between PT-112 sensitivity and the metabolic status of tumor cells.

### 3.2. Cytotoxic Effect of PT-112 and Cisplatin in L929, L929dt and Cybrid Cells and Caspase-3 Activation by PT-112 in Sensitive Cells

**Figure 2:**
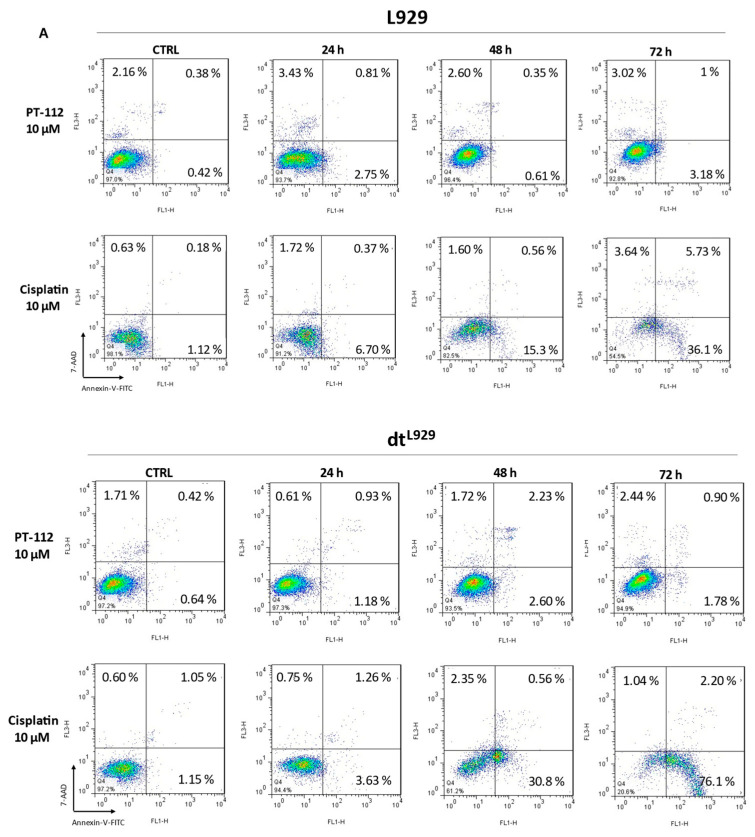

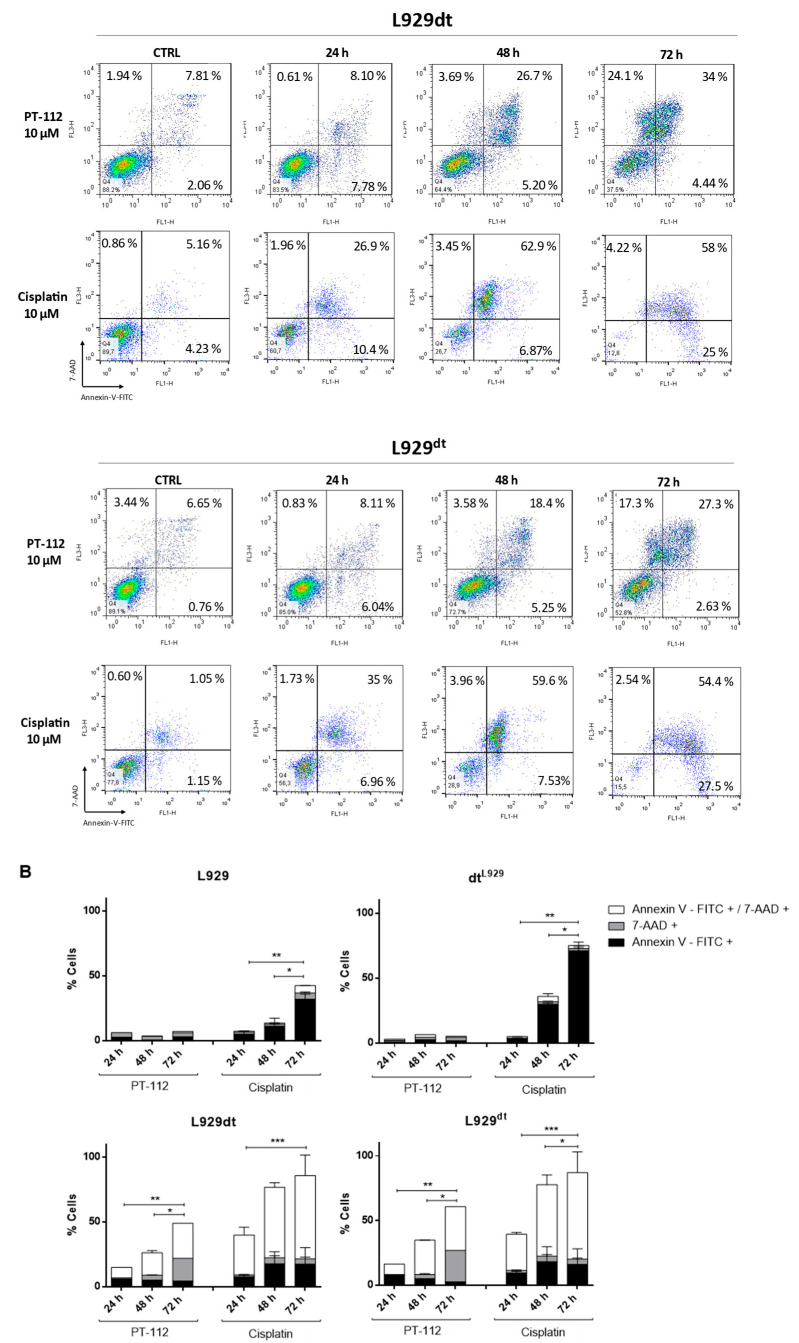
Cytotoxicity of PT-112 or cisplatin. Parental L929, L929dt and cybrids cells were incubated with 10 µM of PT-112 or cisplatin for 24, 48 and 72 h. Then, cells were simultaneously stained with annexin-V-FITC and 7-AAD and analyzed by flow cytometry. (**A**) Dot plots represent the staining evolution of the treated cell population compared to the control. (**B**) Graph bars correspond to a graphical representation of obtained data reporting cell percentage in each quadrant of dot plots. Results are shown as mean ± SD of at least 2 independent experiments performed in duplicate. *, *p* < 0.05; **, *p* < 0.01; ***, *p* < 0.001.

To characterize cell death induction by PT-112 and cisplatin, cells were incubated with 10 µM of PT-112 or cisplatin for 24, 48 or 72 h, and at the end of the incubation, cells were stained with annexin-V-FITC and 7-AAD and analyzed by flow cytometry. The results showed that cisplatin induced cell death in all cell lines, especially after long-time drug exposure (Figure 2). In contrast, PT-112 was cytotoxic only in glycolytic cells, consistent with the differential sensitivity observed in Figure 1 (Figure 2). In cisplatin-induced cell death, the apoptotic cell population (annexin-V^+^, 7-AAD) was observed in all cell lines, albeit cell death was induced more rapidly in the more glycolytic cells (Figure 2B, bar sections colored in black). On the contrary, this population was not appreciably observed at any time point in sensitive L929dt and L929^dt^ cells treated with PT-112. Dead cells (annexin-V^+^, 7-AAD^+^) were detected at an early time point, and the population increased with time (Figure 2B, bar sections colored in white). Finally, at longer exposure times, both glycolytic cell lines showed a population that is positive for 7-AAD and negative for annexin-V staining, typical of necrotic cell death (Figure 2B, bar sections colored in grey). Taken together, these results clearly demonstrate that PT-112 has a distinct mechanism of action from cisplatin and differential sensitivity to the glycolytic phenotype driven by mtDNA mutations.

**Figure 3:**
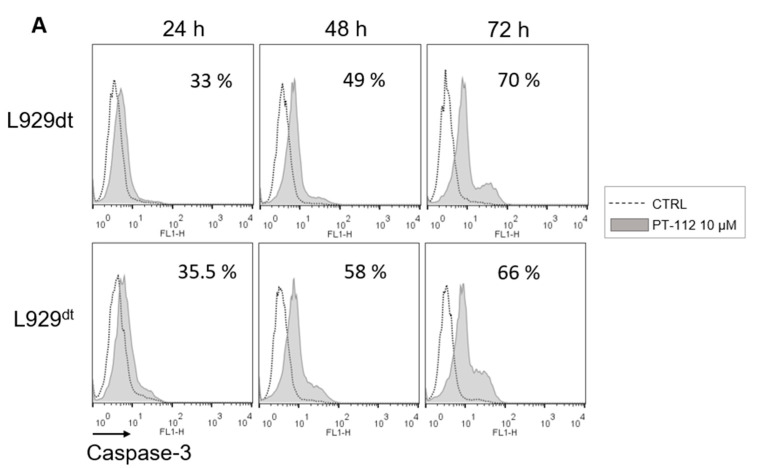

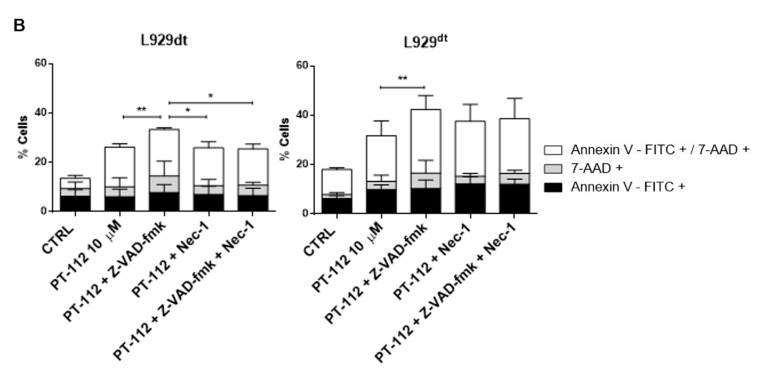
Caspase-3 activation by PT-112 and effect of apoptosis and necroptosis inhibitors. (**A**) Levels of caspase-3 activation upon treatment with PT-112. 3 × 10^4^ cells were treated with 10 µM of PT-112 for 24–72 h. Then, cells were incubated with anti-cleaved caspase-3 labelled with FITC dye and analyzed by flow cytometry. The numbers in each box represent the percentage of cleaved caspase-3 compared to untreated cells. (**B**) Cytotoxicity analysis of PT-112 combined with Z-VAD-fmk and/or necrostatin-1 inhibitors. Cells were pretreated for 1 h with or without pan-caspase or/and necroptosis inhibitors and then, incubated with 10 µM of PT-112 for 48 h. Flow cytometry analysis was performed using annexin-V-FITC and 7-AAD staining. Results are shown as mean ± SD of 3 independent experiments performed in duplicate. Statistical significance is depicted in the graphics. *, *p* < 0.05; **, *p* < 0.01.

To further investigate if and how PT-112 cell death related to the induction of apoptosis in sensitive cells, we analyzed the effect of PT-112 on caspase-3 activation, the main apoptotic executioner. We used a FITC-labelled anti-caspase-3 antibody specific for cleaved, active caspase-3 that can be measured by flow cytometry. As shown in Figure 3A, the levels of active caspase-3 clearly increased in a time-dependent manner in L929dt and L929^dt^ cells sensitive to PT-112-induced cell death. To further investigate the implications of caspase-3 activation in this process, we tested the ability of the general pan-caspase inhibitor Z-VAD-fmk and/or the necroptosis inhibitor necrostatin-1 to prevent cell death induced by PT-112 (Figure 3B). Cells were stained simultaneously with annexin-V-FITC and 7-AAD, and the percentage of the different populations was analyzed by flow cytometry. We observed, in agreement with the results presented in Figure 2A, that PT-112 induced a direct accumulation of double positive (i.e., dead) cells. Z-VAD-fmk, necrostatin-1 or their combination did not inhibit cell death, and the double positive population remained the dominant group in all cases. In fact, cells were more sensitive to PT-112 treatment in the presence of Z-VAD-fmk. Co-incubation with necrostatin-1 prevented this Z-VAD-fmk-induced increase in sensitivity, though this effect was only significant in L929dt cells. These data indicate that neither apoptosis nor necroptosis were the main mechanisms of cell death induced by PT-112 in these cell lines, while a small necroptotic component is observed only if caspases are inhibited. This is reminiscent of other cell death inducers such as TNF-α, specifically in L929 cells [25].

### 3.3. PT-112 Induces Autophagosome Formation

**Figure 4:**
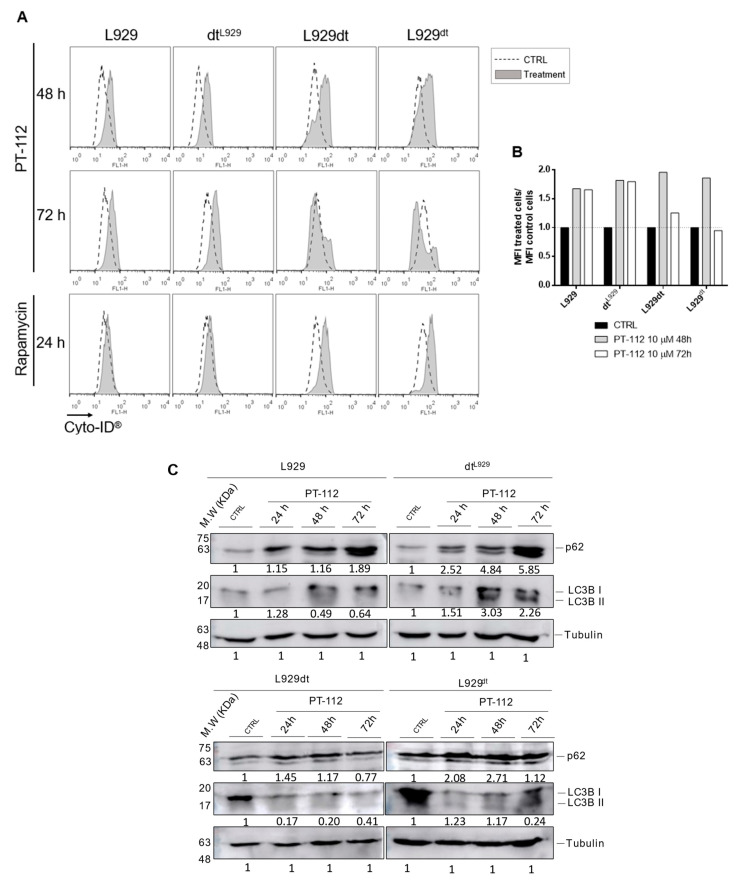
PT-112 induces the initiation of autophagy. (**A**) Analysis of autophagosome formation. Cells were incubated with 10 µM of PT-112 for 48–72 h. The autophagosome formation was analyzed by flow cytometry using the Cyto-ID^®^ method. (**B**) Graphical representation of data obtained with Cyto-ID^®^ analysis. Mean fluorescence intensity (MFI) of treated cells compared to untreated cells is shown. (**C**) Expression levels of p62 and LC3BI/II upon PT-112 treatment. Tubulin was used as a control of protein load. The uncropped blots are shown in Figure S3.

After observing that PT-112 was not reliant on apoptosis or necroptosis (Figure 2 and Figure 3), we tested the possibility that it could induce autophagy. The initiation of autophagy was analyzed using the Cyto-ID^®^ method that allows detection of intracellular autophagosome formation by flow cytometry. As shown in Figure 4, PT-112 clearly induced autophagosome formation in all cell lines at 48 h of PT-112 treatment. At 72 h, autophagosome formation decreased in L929dt and L929^dt^ cells, possibly due to the induction of cell death. On the contrary, in L929 and dt^L929^ cells, autophagosome formation was maintained at 72 h (Figure 4A,B). Of note, we observed that L929dt and L929^dt^ cells were more sensitive to autophagy induction by rapamycin than L929 and dt^L929^ cells (Figure 4A). To further investigate the activation of autophagy upon PT-112 treatment, we analyzed expression levels of p62 and the conversion of LC3BI to LC3BII, known indicators of autophagy induction. The results showed a tendency towards the conversion of LC3BI to LC3BII in L929, and especially in dt^L929^ cells, and a gradual accumulation of p62 (Figure 4C). In L929dt and L929^dt^ cells, we did not observe significant changes in p62 levels, while a rapid reduction in LC3BI levels was observed after drug treatment and accompanied by the appearance of faint LC3BII bands. The Cyto-ID^®^ results, the most sensitive method to detect autophagosome formation [26], and the LC3B data demonstrate that PT-112 initiates the autophagy process. However, the absence of p62 reduction or degradation indicates this process did not reach completion.

The observation of cells on the microscope after treatment with PT-112 showed abundant brilliant spots inside the cytoplasm of all four cell lines, which may correspond to autophagosomes. In addition, in L929dt and L929^dt^ cells sensitive to cell death, induction by small, uniform PT-112 cell debris was also detected (Figure S1).

### 3.4. PT-112 Affects Mitochondrial Membrane Potential and Induces Massive Mitochondrial Reactive Oxygen Species (ROS) Generation in Sensitive Cells

**Figure 5:**
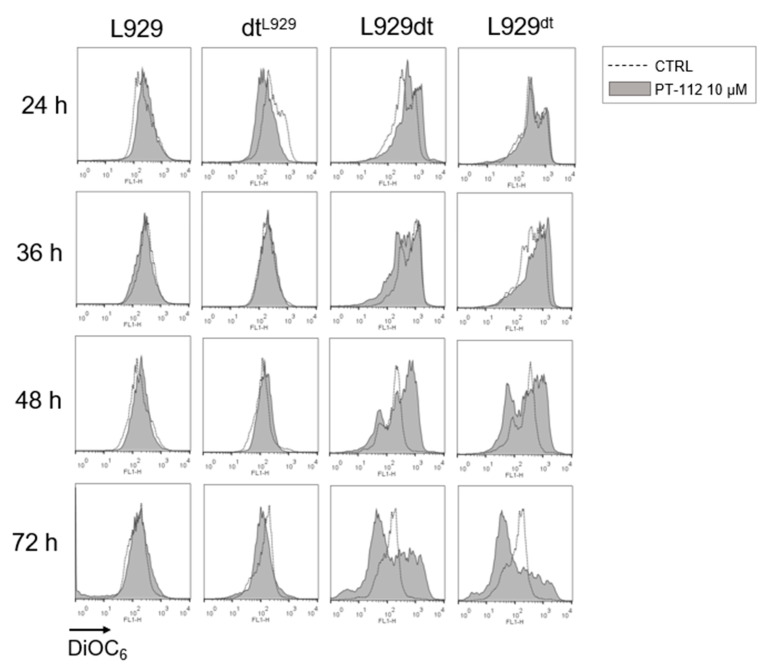
Analysis of mitochondrial membrane potential (ΔΨm) upon treatment with PT-112 at different incubation times. A total of 3 × 10^4^ cells were incubated with 10 µM of PT-112 for 24, 36, 48 and 72 h at 37 °C. Changes in ΔΨm were determined by staining with DiOC_6_ and analyzed by flow cytometry. As shown in the legend, dotted histograms correspond to fluorescence values of untreated cells, and grey-colored histograms correspond to those of treated cells.

One typical event related to the activation of the mitochondrial apoptotic pathway is the loss of mitochondrial membrane potential (ΔΨm) [27]. Hence, we analyzed the effect of PT-112 on ΔΨm using DiOC_6_ (3) staining and flow cytometry. As shown in Figure 5, while ΔΨm did not experience any change during the 72 h incubation with PT-112 in L929 and dt^L929^ cells, a significant change was observed in the PT-112 sensitive, glycolytic cells. Remarkably, ΔΨm increased in these cells upon PT-112 treatment, showing the appearance of a cell population with hyperpolarized mitochondria at 48 h, simultaneously accompanied by a population that partially lost ΔΨm. Both populations were detected at 72 h, but with a larger fraction of cells showing a loss of ΔΨm.

**Figure 6:**
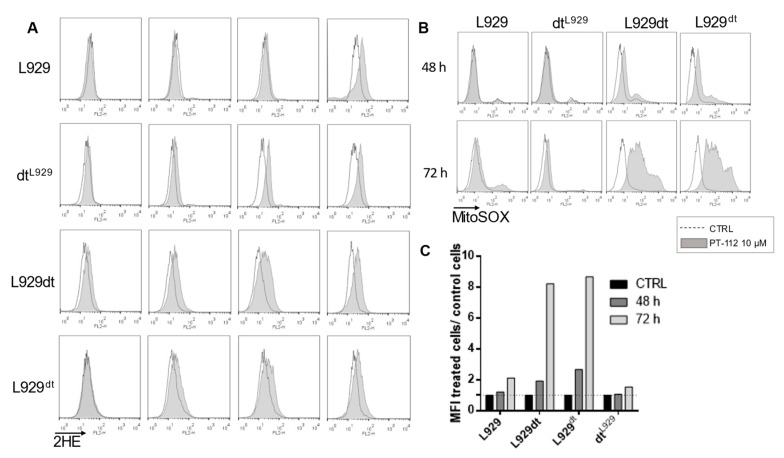
Analysis of total and specific mitochondrial ROS production upon treatment with PT-112 at different incubation times. (**A**) A total of 3 × 10^4^ cells were incubated with 10 µM of PT-112 for 24, 36, 48 and 72 h at 37 °C. Total ROS production was determined by staining with 2HE and flow cytometry. (**B**) Specific mitochondrial ROS production after incubation with 10 µM of PT-112. Cells were stained with a mitochondrial superoxide indicator MitoSOX™ for 15 min at 37 °C, in darkness. The fluorescence intensity values of treated cells compared to control cells (CTRL) were determined by flow cytometry. As shown in the legend, dotted histograms correspond to the fluorescence of untreated cells, and grey-colored histograms correspond to the fluorescence values of treated cells. (**C**) Graphical representation of data obtained in Figure 6B. Data are shown as MFI of treated cells compared to untreated cells.

Next, we studied the effects of PT-112 exposure on ROS production. First, we performed a time-course determination of total ROS generation by detection of 2HE oxidation by flow cytometry. As shown in Figure 6A, we observed a moderate increase in total ROS production in a time-dependent manner in all cell lines tested, reaching maximum levels between 48 and 72 h. L929 and dt^L929^ cells showed similar levels of total ROS production to L929dt and L929^dt^ cells after 72 h of exposure, but the rate of increase in ROS levels was faster in the latter, sensitive cell types. To further delineate these effects, we determined specific mitochondrial ROS (mtROS) production upon PT-112 treatment using the MitoSOX™ reagent. As shown in Figure 6B,C, mtROS production was massively increased in sensitive cells after treatment with PT-112, while little to no change was observed in insensitive L929 or dt^L929^ cells, suggesting that this event may be related to cell death induced by PT-112.

**Figure 7:**
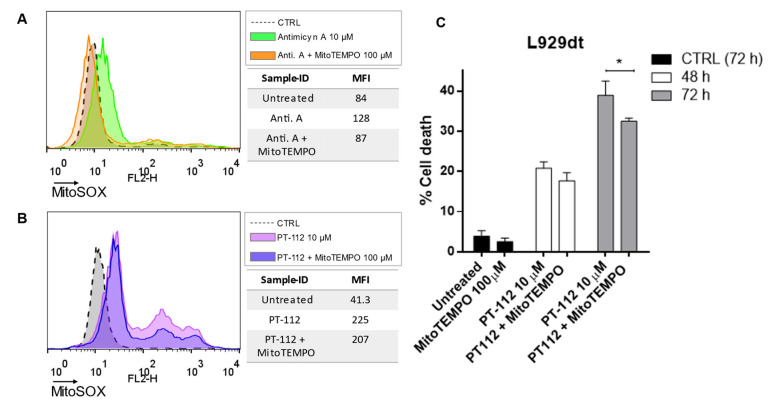
Partial inhibition of PT-112-induced mtROS generation and cell death in L929dt cells by the mtROS scavenger MitoTempo. A total of 3 × 10^4^ cells were seeded in a 96-well plate in phenol red-free medium and some wells were incubated with 100 µM of MitoTEMPO for 2 h. Then, 10 µM of antimycin A or PT-112 were added and incubated for 48 or 72 h, respectively. (**A**,**B**) mtROS levels were measured using MitoSOX™ staining after treatment with either (**A**) antimycin A or (**B**) PT-112. (**C**) Cell death induced by PT-112 in the presence or absence of MitoTEMPO after 48 or 72 h was evaluated by flow cytometry using annexin-V-FITC and 7-AAD staining. Results are shown as mean ± SD of at least 2 independent experiments performed in duplicate. * *p* < 0.05.

Next, we attempted to further investigate the causality of ROS generation in the cell death process induced by PT-112. Since PT-112-induced ROS generation seems to be concentrated in mitochondria, we used the mitochondria-specific ROS scavenger, MitoTEMPO. As shown in Figure 7A, MitoTEMPO was able to almost completely abolish mitochondrial superoxide generation induced by the potent mitochondrial complex III inhibitor, antimycin A. PT-112 induced a higher amount of mitochondrial superoxide anion compared to antimycin A, and co-incubation with MitoTEMPO resulted in a limited reduction in mitochondrial ROS (Figure 7B). A reduction in PT-112-induced L929dt cell death was also observed when co-incubated with mitoTEMPO and was statistically significant after 72 h (Figure 7C). These data suggest that mitochondrial ROS generation is implicated in PT-112-induced cell death, although it is experimentally difficult to prevent ROS formation in order to finitely determine causality by chemical means.

As an alternative approach, we decided to use L929-ρ^0^ cells. ρ^0^ cells are devoid of mtDNA by prolonged exposure to ethidium bromide and are unable to perform OXPHOS or generate mitochondrial ROS; although, upon specific treatments (such as with perforin/granzyme B), they can generate ROS from extra-mitochondrial sources [17,28]. We tested the growth inhibition effect of PT-112 and cisplatin on these L929-ρ^0^ cells in order to assess whether the complete absence of mitochondrial function would affect PT-112 sensitivity.

**Figure 8:**
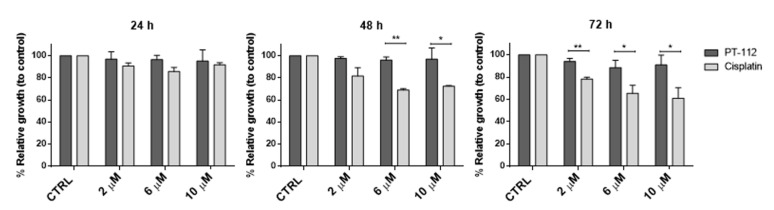
Cell growth analysis after treatment of L929-ρ^0^ cells with PT-112 or cisplatin. Cells were treated with increasing concentrations of PT-112 or cisplatin, incubated for 24–72 h, and relative growth was measured by MTT assay. Results correspond to the percentage of relative growth compared to untreated, control cells (CTRL). Results are shown as mean ± SD of at least 2 independent experiments performed in duplicate. * *p* < 0.05, ** *p* < 0.01.

As shown in Figure 8, while cisplatin inhibited the growth of these cells, PT-112 scarcely affected their growth rate at any concentration or time of incubation. In addition, while PT-112 did not robustly induce cell death in parental L929 cells (Figure 2), it did inhibit the growth of these cells to some extent (Figure 1), contrasting with the lack of effects on the growth of L929-ρ^0^ cells. These differences in PT-112 effects between L929 and the L929-ρ^0^ cells are not due to differences in the basal growth rate of these two types of cells. These data suggest that the effects on mitochondria play a large role in the PT-112 mechanism of action, potentially mediated via mtROS generation.

### 3.5. Effect of PT-112 on Mitochondrial CoQ10 Levels

**Figure 9:**
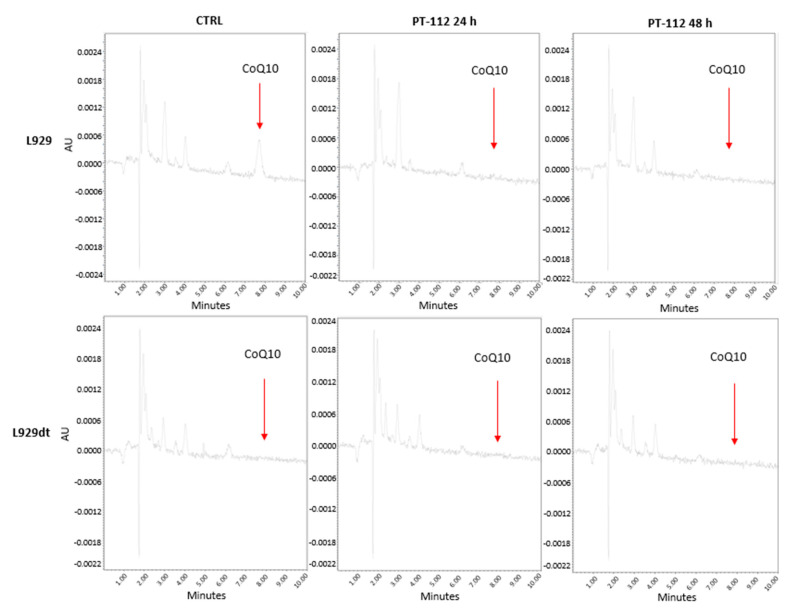
HLPC determination of mitochondrial CoQ10 amounts on L929 or L929dt cells after treatment with PT-112. The mitochondrial lipid fraction of 20 × 10^6^ cells (L929 or L929dt), treated with 10 µM PT-112 for 24 or 48 h was extracted. Untreated cells (CTRL) were also included. CoQ10 levels were determined by HPLC methodology (see Materials and Methods). Commercial CoQ10 was used as a positive control (see Figure S2). Representative plots are shown.

The mevalonate pathway not only provides farnesyl or geranylgeranyl units for protein post-translational modifications but also provides longer prenyl groups for the final steps of Coenzyme Q synthesis, generating coenzyme Q9, Q10 or longer ubiquinone derivatives [29,30]. In all these steps of the mevalonate pathway, pyrophosphate derivatives are central to enzyme activity, and we speculated that it is possible PT-112 could act on these enzymes through its pyrophosphate moiety. Hence, we decided to study the possible effect of PT-112 on CoQ10 levels, analyzed by HPLC determination in mitochondrial lipid extracts. We first optimized the method of detection using commercial CoQ10. We confirmed the purity of commercial CoQ10 and determined its absorbance peak at 274.9 nm (Figure S2, right panel). We then determined the retention time of pure CoQ10 in the HLPC column to be used in the analysis of cell samples, obtaining a retention time of 7.749 min (Figure S2, left panel). When we analyzed mitochondrial lipid extracts using this HPLC column, we observed that PT-112 substantially reduced the amount of CoQ10 in L929 mitochondria (Figure 9). However, these cells, which are OXPHOS-competent, were not sensitive to PT-112-induced cell death, although their growth was inhibited by 40% after 72 h of exposure (see Figure 1). In L929dt cells that are sensitive to PT-112-induced cell death, the basal amount of CoQ10 was below detection.

### 3.6. PT-112 Induces CRT Exposure on the Surface of Sensitive Cells

**Figure 10:**
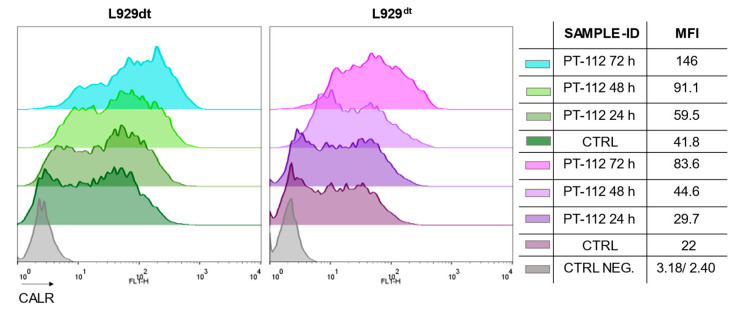
CRT exposure upon PT-112 treatment. Representative plots are shown. CRT exposure was analyzed in L929dt or L929^dt^ cells upon treatment with 10 μM PT-112 for the time indicated. Grey histograms correspond to the labeling with the secondary antibody alone and are considered as negative controls (CTRL NEG.), while control labelings correspond to the basal labeling of untreated cells (CTRL). Representative plots are shown.

PT-112 has been shown to induce ICD, characterized by CRT exposure on the cell membrane, together with emission of other danger signals in several types of tumor cells [7]. Hence, we tested whether PT-112 would induce ICD in sensitive L929dt and L929^dt^ cells. As shown in Figure 10, PT-112 induced CRT exposure on the surface of both cell lines. In addition, the level of CRT exposure increased with time of treatment. This finding, together with the spreading of cell debris observed upon PT-112 treatment as shown in Figure S1, demonstrates the high immunogenic potential of PT-112-induced cell death in this cellular system.

### 3.7. Cells Sensitive to PT-112 Express High Levels of HIF-1α

**Figure 11:**
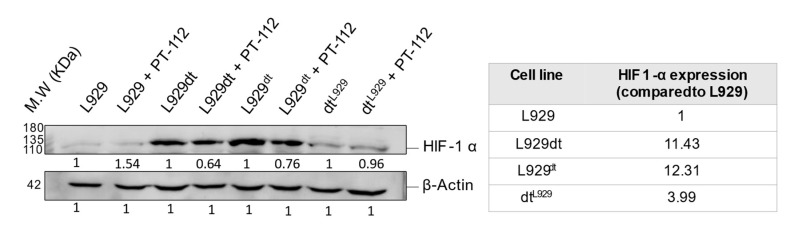
Analysis of HIF-1α expression levels in the presence or absence of PT-112. Cells were incubated with 10 µM of PT-112 for 72 h. Cell lysates were resolved in an SDS-PAGE 6% polyacrylamide gel, and proteins were transferred to nitrocellulose membrane and incubated with a specific antibody against HIF-1α. β-actin was used as a control of the protein loaded. The table shows the percentage of protein expression in basal conditions compared to parental L929 cells. The uncropped blots are shown in Figure S3.

L929dt and L929^dt^ cells favor glycolysis for ATP production due to their defective mitochondria associated with mtDNA mutations, as described [18]. Since HIF-1α is a transcription factor that regulates the expression of key genes implicated in glycolysis [31], we decided to further investigate the relationship between the metabolic profile of L929dt and L929^dt^ cells and basal HIF-1α expression in our cellular models and any changes observed after treatment with PT-112. As shown in Figure 11, we demonstrated that even in the presence of oxygen, L929dt and L929^dt^ cells expressed elevated levels of HIF-1α compared to L929 and dt^L929^ cells (around a 12-fold increase compared with parental L929 cells). PT-112 did not substantially affect expression levels of HIF-1α across all cell lines, yet higher baseline expression was found in those cells more sensitive to PT-112. These data suggest that sensitivity to PT-112 could be closely related to HIF-1α expression and could have prognostic and clinical applications.

## 4. Discussion

PT-112 presents the intriguing possibility that an anti-cancer agent with a platinum chiral center may operate through a mechanism of action that is selective to cancer metabolic processes, specifically mitochondrial dysfunction. In contrast to the canonical understanding of the cell death mechanism of approved platinum salts, it has been shown previously that PT-112 minimally binds nuclear DNA [1,4]. Structurally, PT-112 is unique because it contains a pyrophosphate moiety, which among other attributes contributes to a marked osteotropism, something that the closest structural analog, oxaliplatin, does not share [1].

Beyond osteotropism, however, based upon the data presented herein, it is possible that PT-112’s pyrophosphate component may, in fact, be associated with its cell death mechanisms, including effects on cancer metabolic pathways. Examples of active anabolic pathways in tumor cells are the pentose phosphate pathway, needed for the synthesis of DNA and RNA nucleotides [32], and the mevalonate pathway, needed for the de novo synthesis of sterols and geranyls [33]. Farnesyl and geranylgeranyl backbones are required for the post-translational modification of relevant proteins in signaling, such as Ras [30], and also for the synthesis of mitochondrial coenzyme Q derivatives [29]. Notably, both pathways involve pyrophosphates for specific enzyme activities. This subject matter has not been deeply studied in the field of cancer treatment, perhaps because there are no approved drugs that contain pyrophosphate groups. Potential pyrophosphate-driven effects could include an increase in PT-112 uptake by tumor cells that are especially metabolically active and dependent on the mevalonate or the pentose phosphate pathway and/or PT-112 more directly affecting those pathways. Further studies will be needed to test these hypotheses.

L929dt and L929^dt^ cybrid cells present mutations in mtDNA, causing a shift towards a glycolytic phenotype. These cells were especially sensitive to cell death induced by PT-112 while tumor cells with an intact OXPHOS pathway (L929 and dt^L929^ cybrid cells) were shown to be less sensitive to PT-112 and, strikingly, to lack a dose-response. In contrast, the classical Pt-containing drug cisplatin induced cell death and a dose-response across all the cell lines, irrespective of their metabolic and mitochondrial status. In addition, while cisplatin follows the canonical apoptotic pathway used by many chemotherapeutic drugs such as doxorubicin [27,34], PT-112 does not appear to comply with this canonical pathway, rather showing hints of necrotic cell death. Moreover, while PT-112 activates caspase-3 at the same time as cell death, the general caspase inhibitor Z-VAD-fmk does not inhibit PT-112-induced cell death, alone or in combination with the necroptosis inhibitor necrostatin-1. On the other hand, PT-112 induced the initiation of autophagy in all cell lines, detected by the Cyto-ID^®^ method and by an increase in the LC3BII/LC3BI ratio, although it appears that the autophagy process was not completed, as evidenced by the lack of p62 degradation.

The observed difference in PT-112 sensitivity based on the baseline metabolic and mitochondrial mutational status of the L929 model cells led us to investigate the possibility that PT-112 cell death effects could themselves involve mitochondrial pathways. Indeed, PT-112 induced ROS in all cells tested, regardless of their sensitivity to cell death induction, although ROS appeared more rapidly in more sensitive cells. Importantly, when this analysis was restricted to the detection of mtROS, only the PT-112-sensitive, OXPHOS deficient cells showed a massive mtROS accumulation. We demonstrated partial protection from PT-112-induced cell death in sensitive cells by the use of the mitochondria-restricted ROS scavenger MitoTEMPO. Moreover, we have shown that L929-ρ^0^ cells, devoid of mtDNA and unable to perform OXPHOS or to generate mtROS [17], were less sensitive to PT-112-induced growth inhibition versus the parental L929 cell line. These data point to the observed massive mitochondrial ROS generation as an important event in PT-112-induced cell death. It should be noted that, although L929-ρ^0^ cells are an extreme and unnatural case of the glycolytic phenotype, their inability to generate mitochondrial ROS makes them highly resistant to PT-112, reinforcing the importance of this biochemical event in PT-112-induced cell death. On the other hand, this observation indicates that in our experimental system, the sensitivity to PT-112 is associated more specifically with the presence of defective mitochondria that generate ROS upon stress and not generally with every glycolytic phenotype.

PT-112 also affected mitochondrial membrane potential (ΔΨm) in sensitive cells, albeit in an unexpected way. After short incubation times with PT-112 (24–36 h), an initial mitochondrial hyperpolarization was observed. At later time points (48 h), two cell populations were detected: one with hyperpolarized mitochondria and another one that showed loss of ΔΨm. At 72 h, when cell death notably increased in response to PT-112, the population with a loss of membrane polarization predominated.

Based on the literature surrounding bisphosphonates [35,36] and the structural similarity between bisphosphonates and pyrophosphates, it is possible that PT-112 could act directly on enzymes of the mevalonate pathway, such as farnesyltransferase or geranylgeranyl transferase. Increases in farnesyltransferase expression activity have been reported in prostate cancer patients, correlating with poor prognosis [37], and PT-112 has shown some efficacy signals in late-stage mCRPC, either alone [13] or in combination with PD-L1 immune checkpoint inhibition [14]. In support of such a hypothesis, we note that Qiu et al. [36] have developed [Pt(en)]_2_ZL, a complex which conjugates the bisphosphonate zoledronic acid with Pt^2+^ ions and demonstrated that it prevented the prenylation of small G proteins through inhibition of the mevalonate pathway.

The mevalonate pathway not only provides farnesyl or geranylgeranyl units for protein post-translational modifications but also provides longer prenyl groups for the final steps of Coenzyme Q synthesis, generating coenzyme Q9, Q10 or longer ubiquinone derivatives [29,30]. In all these steps of the mevalonate pathway, pyrophosphate derivatives are important for enzymatic activities, and PT-112 could act on these enzymes through its pyrophosphate moiety. We have demonstrated that PT-112 substantially reduced the amount of CoQ10 in L929 mitochondria. Although L929 cells, which are OXPHOS-competent, are not sensitive to PT-112-induced cell death, their growth is substantially reduced by the drug, and this could be related to this CoQ10 depletion. In L929dt cells that are sensitive to PT-112-induced cell death, the basal amount of CoQ10 is much lower than in L929 cells (i.e., below the detection level). The low amount of CoQ10 in these cells, as well as the mtDNA mutations in complex I components, could explain the massive accumulation of mitochondrial ROS observed upon PT-112 treatment, the resulting non-apoptotic form of cell death, and evidence of ICD. Additional studies will be needed to better understand the relevance of CoQ10 in PT-112’s anti-cancer activity and explore if these effects originate from effects on the mevalonate or other biological pathways.

Our data point to a cell death mechanism in which PT-112 would not kill cells by apoptosis nor by necroptosis but rather by inducing the initiation of an unresolved autophagy process, possibly leading to ER stress, together with the above-mentioned massive mtROS generation. It is also interesting to note that autophagy processes, normally not related to cell death, are associated with immunogenic signals and damage-associated molecular pattern (DAMP) emission [38]. In line with this conceptual approach, we demonstrated that PT-112 induces CRT exposure in sensitive L929dt and L929^dt^ cells, a *bona fide* marker of ICD. These data correlate with prior work in vitro and in vivo showing that PT-112 causes ICD [7].

Finally, we demonstrate that the expression of HIF-1α is higher in cells that are sensitive to PT-112 compared to cells with an intact OXPHOS pathway. HIF-1α is the master regulator of the glycolytic phenotype and is caused by hypoxia or by aerobic glycolysis observed in many tumor cells [31]. Our data indicate that this relationship is conserved in our cellular model, in which the glycolytic phenotype originates from defective mitochondrial function due to mtDNA mutations [18]. In addition, low levels of CoQ10, as detected in L929dt cells at the basal state, have been recently correlated with high HIF-1α expression and stabilization [39]. Based on these observations, HIF1-α expression may be associated with differential sensitivity to PT-112, warranting further studies to explore future clinical applications.

## 5. Conclusions

PT-112 is a novel pyrophosphate-Pt conjugate with favorable safety profiles and evidence of efficacy in Phase I clinical trials. Using the L929 murine in vitro model system, we demonstrated that tumor cells presenting mtDNA mutations and the resulting glycolytic phenotypes (L929dt and L929^dt^ cybrid cells) were more sensitive to cell death induced by PT-112 compared to the parental and cybrid cells with an intact OXPHOS pathway (L929 and dt^L929^ cybrid cells). PT-112 caused an incomplete autophagic process, along with massive accumulation of mtROS. PT-112 also reduced the amount of mitochondrial CoQ10 in L929 cells, while the basal CoQ10 levels were below our detection limits in L929dt cells, suggesting a potential relationship between a low basal level of CoQ10 and PT-112 sensitivity. In addition, PT-112 induced CRT exposure in sensitive L929dt and L929^dt^ cells, consistent with ICD. Finally, the expression of HIF-1α was much higher in glycolytic cells sensitive to PT-112 compared to cells with an intact OXPHOS pathway. Taken together, these findings suggest the selectivity of PT-112 to differential metabolic statuses of cancer cells, with the potential for clinical applications of PT-112 in metabolically aggressive cancers.

## 6. Patents

The use of PT-112 to treat cancer is protected by US patent 9,688,709, registered on 27 June 2017, and an application has been submitted pertaining to the identification of PT-112-sensitive cancer cells on the basis of glycolytic features (international application number PCT/US2021/055907).

## Data Availability

The datasets generated and/or analyzed during the current study are available from the corresponding authors on reasonable request.

## Supplementary Materials

The following supporting information can be downloaded at: https://www.mdpi.com/article/10.3390/cancers14163851/s1, Figure S1: Cell morphology after PT-112 treatment. Figure S2: HPLC chromatogram and absorbance spectrum of commercial CoQ10. Figure S3: Uncropped blots shown in the Figures of the manuscript.
