# Supplementary material for: PT-112 Induces Mitochondrial Stress and Immunogenic Cell Death, Targeting Tumor Cells with Mitochondrial Deficiencies
Source: Cancers (Basel). 2022 Aug 9;14(16):3851. doi: 10.3390/cancers14163851 (PMC9405950; doi:10.3390/cancers14163851)
Supplement: Supplementary file 1 [file cancers-14-03851-s001.zip › cancers-1807659-supplementary.pdf]

Supplementary material

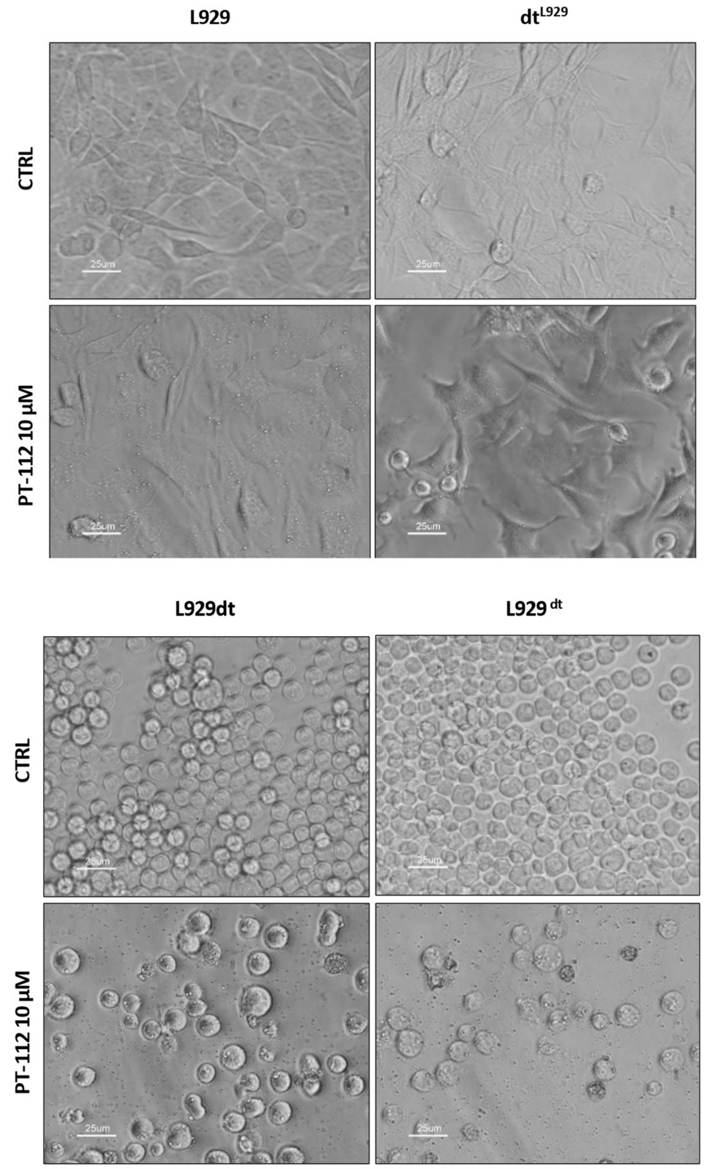

Figure S1. Cell morphology after PT-112 treatment. Phase-contrast micrographs of control cells (CTRL) and cells treated or not (CTRL) with 10 µM PT-112 for 72h.

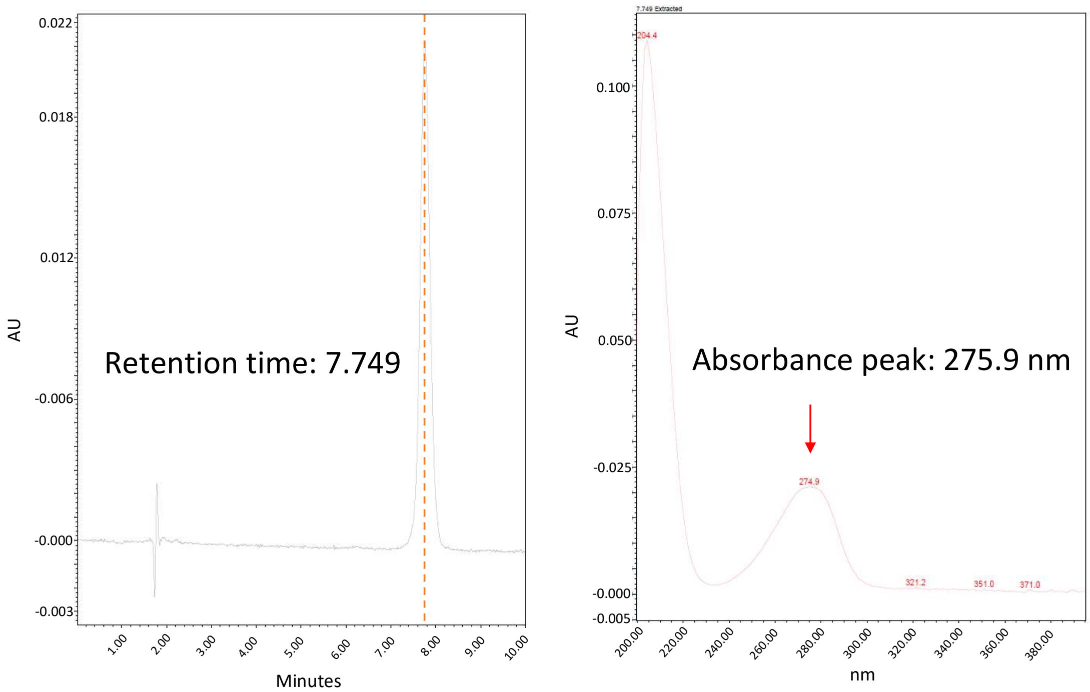

Figure S2. HPLC chromatogram (left panel) and absorbance spectrum of commercial CoQ10 (right panel). Concentrated sample of commercial CoQ10 was prepared and analyzed by HPLC. Retention time (7.749 min) and absorbance peak (274.9 nm) were used as a reference in order to identify the CoQ10 peak in the samples.

Figure S3. Uncropped blots shown in the Figures of the manuscript.

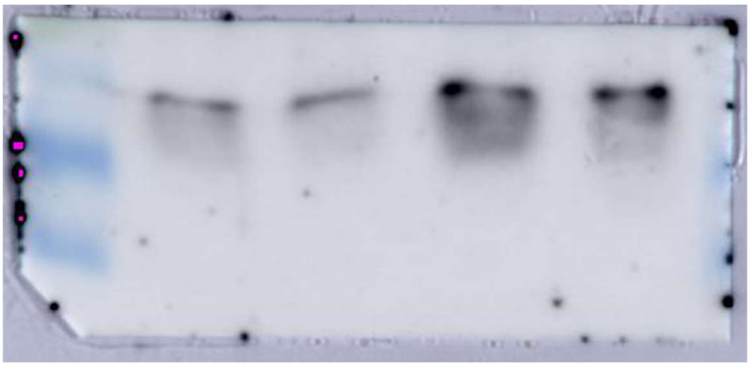

Anti-LC3BI/LC3BII immunoblot on L929 cells shown in Figure 4C

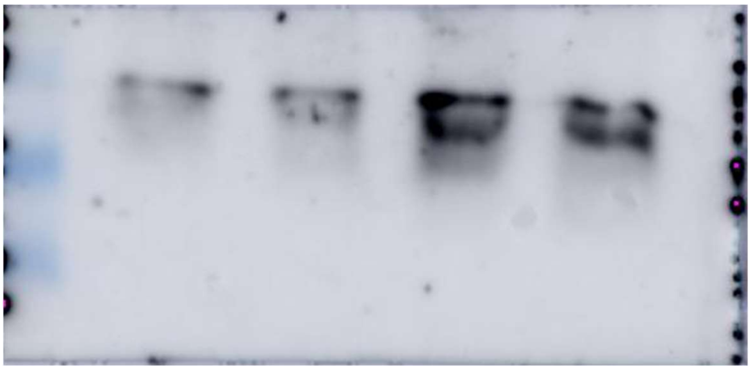

Anti-LC3BI/LC3BII immunoblot on dt[L929] cells shown in Figure 4C

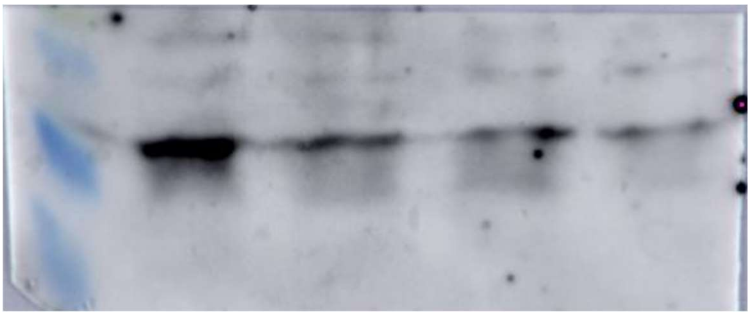

Anti-LC3BI/LC3BII immunoblot on L929dt cells shown in Figure 4C

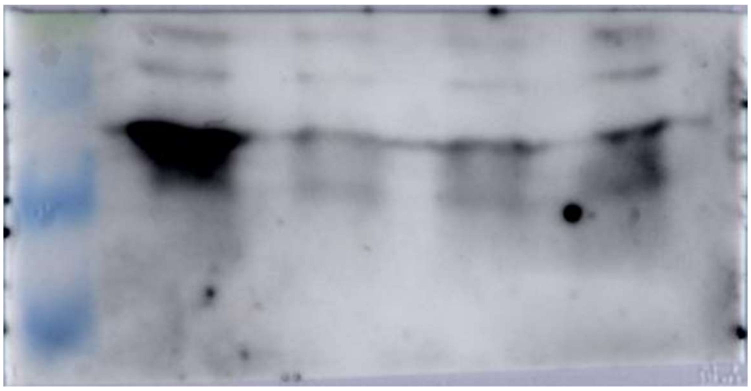

Anti-LC3BI/LC3BII immunoblot on L929[dt] cells shown in Figure 4C

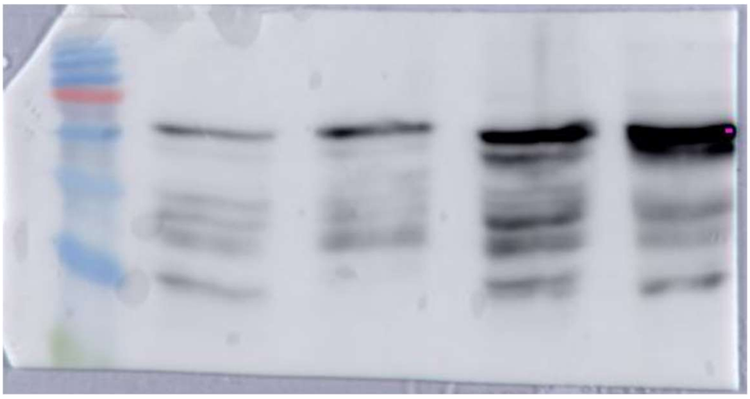

Anti-p62 immunoblot on L929 cells shown in Figure 4C

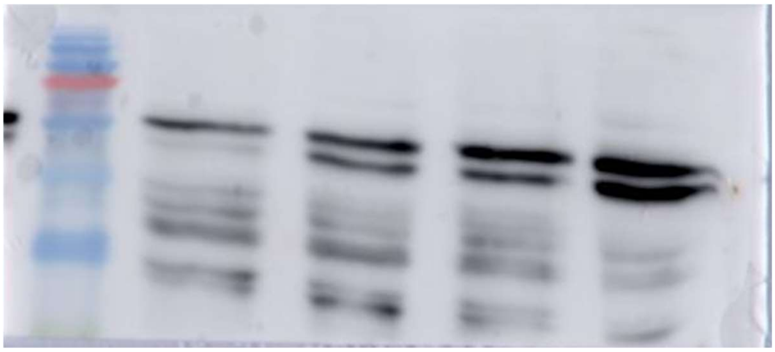

Anti-p62 immunoblot on dt^{L929} cells shown in Figure 4C

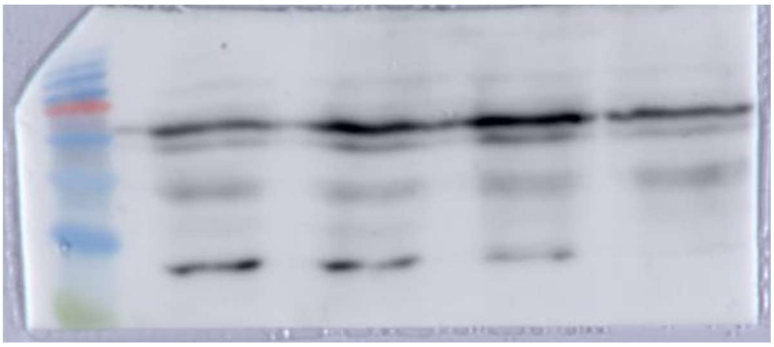

Anti-p62 immunoblot on L929dt cells shown in Figure 4C

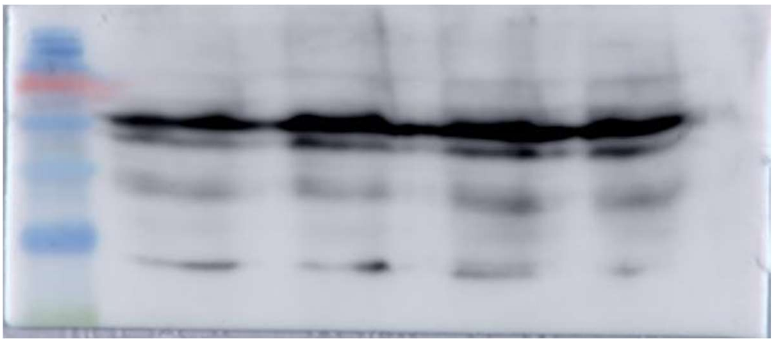

Anti-p62 immunoblot on $L929^{dt}$ cells shown in Figure 4C

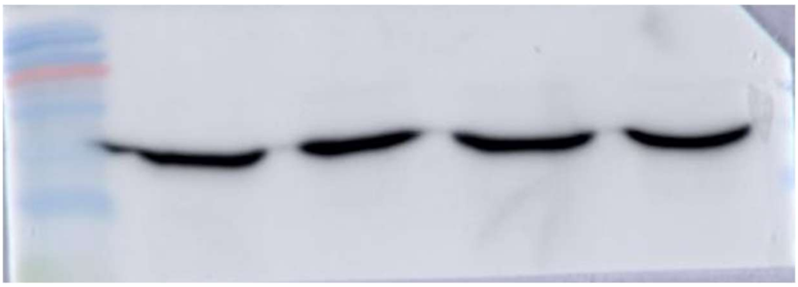

Anti-tubulin immunoblot on L929 cells shown in Figure 4C

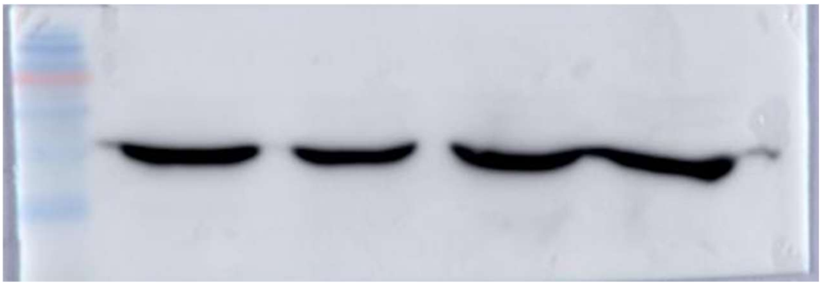

Anti-tubulin immunoblot on dt^{L929} cells shown in Figure 4C

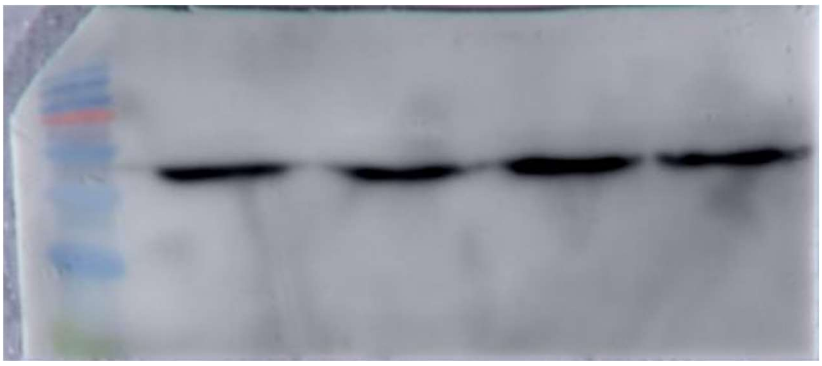

Anti-tubulin immunoblot on L929dt cells shown in Figure 4C

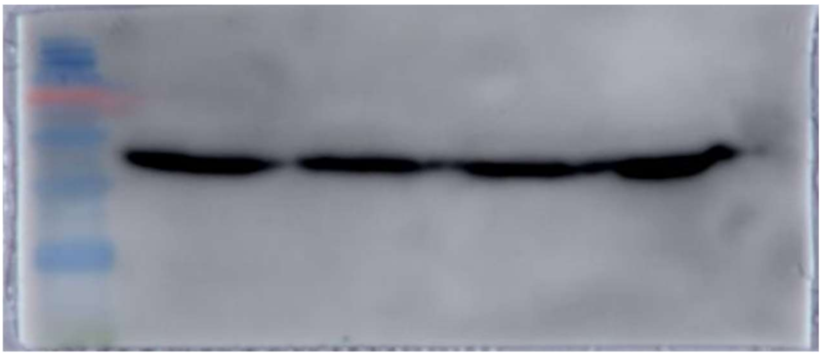

Anti-tubulin immunoblot on L929^{dt} cells shown in Figure 4C

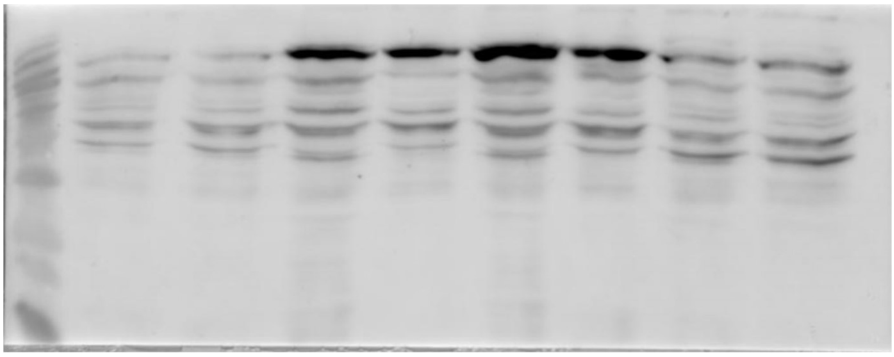

Anti-HIF-1α immunoblot shown in Figure 11

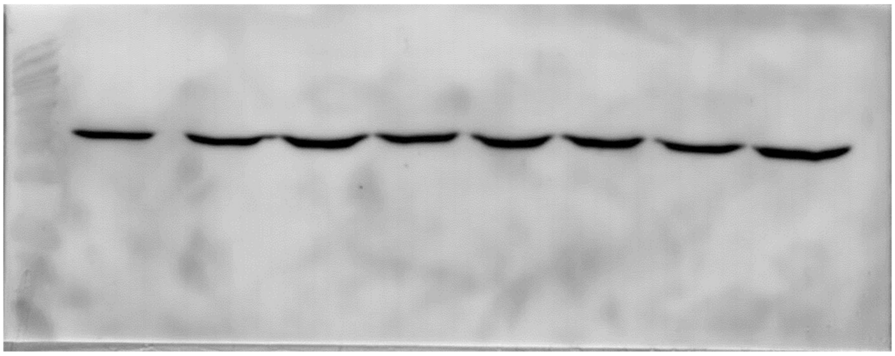

Anti–β–actin immunoblot shown in Figure 11
